# Prospects of COVID-19 Vaccination in Romania: Challenges and Potential Solutions

**DOI:** 10.3389/fpubh.2021.644538

**Published:** 2021-02-10

**Authors:** Stefan Dascalu, Oana Geambasu, Ovidiu Covaciu, Razvan Mircea Chereches, Gabriel Diaconu, Gindrovel Gheorghe Dumitra, Valeriu Gheorghita, Emilian Damian Popovici

**Affiliations:** ^1^Department of Zoology, University of Oxford, Oxford, United Kingdom; ^2^Avian Influenza Group, The Pirbright Institute, Pirbright, United Kingdom; ^3^Harvard T.H. Chan School of Public Health, Harvard University, Cambridge, MA, United States; ^4^Division of Infectious Diseases and Tropical Medicine, University Hospital, Ludwig Maximilian University of Munich, Munich, Germany; ^5^Healthy Romanian Coalition, Bucharest, Romania; ^6^Department of Public Health, Babes-Bolyai University, Cluj-Napoca, Romania; ^7^Mindcare Centre for Excellency in Psychiatry and Psychotherapy, Bucharest, Romania; ^8^Romanian National Society of Family Medicine, Bucharest, Romania; ^9^Romanian COVID-19 Vaccine Strategy Committee, Bucharest, Romania; ^10^Central Military Emergency Hospital, Carol Davila University of Medicine and Pharmacy, Bucharest, Romania; ^11^Department of Epidemiology, Victor Babes University of Medicine and Pharmacy, Timisoara, Romania

**Keywords:** COVID-19, Romania, vaccination, communication, public health

## Abstract

The rapid advancement in vaccine development represents a critical milestone that will help humanity tackle the COVID-19 pandemic. However, the success of these efforts is not guaranteed, as it relies on the outcomes of national and international vaccination strategies. In this article, we highlight some of the challenges that Romania will face and propose a set of solutions to overcome them. With this in mind, we discuss issues such as the infrastructure of vaccine storage and delivery, the deployment and administration of immunisations, and the public acceptance of vaccines. The ways in which Romanian society will respond to a national COVID-19 vaccination campaign will be contingent on appropriate and timely actions. As many of the problems encountered in Romania are not unique, the proposed recommendations could be adapted and implemented in other countries that face similar issues, thereby informing better practices in the management of the COVID-19 pandemic.

## Introduction

The COVID-19 pandemic has generated unprecedented efforts in the development of potential vaccines against its causative agent, SARS-CoV-2 (1, 2). As the entire world welcomes the approval of various COVID-19 vaccines that have successfully completed clinical trials, it is paramount to acknowledge that their implementation will pose major challenges not only on a global scale, but also at the regional and local levels. In the European Union (EU), considerable efforts have been concentrated on ensuring the financial and logistic resources necessary for the production, acquisition, and distribution of COVID-19 vaccines as they become available. As the outcomes of national vaccination campaigns are often variable both among and within EU member states, potential issues will only be fully solved by considering their specific manifestations in each country and region. Moreover, this needs to be done in a timely manner, thereby ensuring that as COVID-19 vaccines become available, their implementation follows as smoothly and efficiently as possible. In this article, we highlight the urgent need for an evidence-based vaccination campaign in Romania. We begin by identifying how some of the main issues concerning vaccination may manifest themselves within the country. We then propose actions which need to be taken during the national COVID-19 immunization campaign in order to maximize its chances of success.

## Challenges Concerning Vaccination in Romania

According to the latest EU evaluation, the Romanian healthcare system has the lowest GDP expenditure toward healthcare, the highest percentage of treatable causes of mortality, and one of the lowest vaccination coverages among member states (3, 4). Indeed, the effects of the underfunded healthcare system were felt immediately during the acute stages of the pandemic. These included, but were not limited to, shortages of appropriate equipment, the understaffing of healthcare units, an inadequate capacity for contact tracing and isolation/quarantine, and a partial public mistrust of the authorities' ability to meet the needs of the population (5). By contrast, the problems concerning COVID-19 vaccination became apparent as the national immunization campaign unfolded. Indeed, in Romania, major challenges had been expected regarding the deployment, distribution, and administration of COVID-19 vaccines (1, 2, 5).

## Distribution Chains and the Delivery of Immunization

The adequate transport and storage of COVID-19 vaccines will be crucial for the success of any national vaccination campaign. However, considering past experiences with other vaccine-preventable infectious diseases, these logistic aspects remain a major impediment in Romania. For example, in 2009, the insufficient supply of MMR vaccine led to only 53 and 43% of children being immunized in November and December, respectively (6). Similarly, in the 2019–2020 flu season, the stock of the influenza vaccine was not enough to meet national demands (7). Hence, an important challenge that Romania has been facing is how to use an infrastructure which is already stretched to its limits. Furthermore, some of the COVID-19 vaccines impose additional logistic difficulties, as they have very stringent requirements in terms of storage and transport (e.g., cold chains of −80°C) (2). However, the transfer of vaccination logistics to the Romanian Ministry of National Defense was able to overcome some of these issues (8). Moreover, as opinion polls indicate that as many as 70% of Romanians place a high degree of trust in the military, we suggest this coordination may heighten the acceptance of a national vaccination campaign (9).

Similarly, for an efficient delivery of any vaccine, an accurate and up-to-date electronic health record is required. In Romania, the database co-opted for the immunisations against COVID-19 was the National Electronic Registry for Vaccinations (RENV). Prior to the COVID-19 pandemic, RENV required all childhood immunisations to be recorded by medical personnel, thus having the prerequisite attributes to serve as a useful tool in any national vaccination campaign (6). Indeed, this system was adapted and extended to include adult COVID-19 vaccinations, thereby acting as a centralized electronic database for monitoring vaccine stocks, the vaccination coverage rate, and adverse reactions post-immunization.

## Prioritization of Vaccine Recipients

One of the recurrent topics worldwide concerned the question of who will be the first beneficiaries of COVID-19 immunizations (1). In Romania, while the prioritization of first responders was understandable due to their significant exposure, authorities needed to employ efficient communication concerning the subsequent priority groups that were to receive the vaccine. As a degree of mistrust was already present with regards to the provision of healthcare services, the authorities had to reassure the public and explain that the prioritization of COVID-19 vaccination was made based on objective and ethically justified criteria (5).

Uncontrolled distribution and reports of individuals “jumping the queue” have the potential to generate mistrust and cause public unrest. Indeed, there are numerous instances of this phenomenon occurring throughout the Romanian healthcare system, as waiting lists are vulnerable to corruption (10). As such, the concern that some individuals can accrue health benefits to the detriment of those less fortunate needs be addressed with complete transparency. Resolving this issue remains paramount for the COVID-19 vaccination programme to succeed in Romania, as it is often invoked by supporters of various conspiracy theories or the anti-vaccination movement (6). Indeed, transparency and clear communication will also serve as the main pillars of educational campaigns aimed at improving public trust in vaccines.

## Raising Awareness and Acceptance of Vaccines

Public outreach concerning immunization is critical for the success of a national COVID-19 vaccination campaign due to several issues (1, 2). For example, the development of current vaccine candidates was much more accelerated in comparison to that of other vaccines in use. Therefore, communication efforts will be continuously required to emphasize that no compromises were made in terms of safety or efficacy. Similarly, as some vaccines use novel technologies (e.g., mRNA), thorough and accessible information needs to be made available to the public. These issues will also allow for anti-vaccine sentiments to develop, and thus appropriate communication strategies will need to be devised to address this problem.

In Romania, traditional media channels such as television or radio were initially used to broadcast material about COVID-19 vaccination. Moreover, the early stages of the campaign included the creation of an official online platform (www.vaccinare-covid.gov.ro) which provided accessible information, including other verified national and international resources (11). However, the results of these communication efforts would be greatly enhanced if information were delivered in a manner more targeted toward specific population subgroups (6). Indeed, any vaccination campaign in Romania will have to overcome the challenges posed by the cultural, socioeconomic, and historical factors specific to the country.

An elaborate, evidence-based educational campaign for raising vaccine acceptance is urgently required. These actions will also need to consider the social desirability factor (the “silent factor”), in the sense that some people may wish to be immunized, but would not disclose that in polls or surveys (12). This phenomenon may be especially relevant if any survey is carried out to assess the vaccine acceptance of a particular population subgroup or in a specific region. Additionally, funds will be needed to address the issues of vaccine hesitancy and refusal in a systematic way, ranging from educating the public to having open debates and providing relevant information about COVID-19 vaccines and the act of immunization itself. Recent national and international surveys (13, 14) troublingly suggest that at least 1 in 3 Romanians would refuse any form of vaccination against COVID-19. By contrast, fewer than the same number of individuals have the intention to immunize themselves if a vaccine becomes available. As many Romanians place safety concerns among the primary reasons for refusing a potential COVID-19 vaccine, strong and transparent communication on the known safety profile of the COVID-19 vaccines will be needed. Therefore, public repositories regarding potential side-effects and clear mechanisms to report such issues are essential for a national vaccination campaign to succeed.

## Combating the Spread of Misinformation

Throughout the COVID-19 pandemic, containing the spread of misinformation was perhaps at least as difficult as controlling new infections. Indeed, Romania is no exception to this phenomenon, and there are numerous instances where public health efforts were hindered by false information. For example, during the early stages of the pandemic, returning members of the Romanian diaspora were vilified on various social media platforms (5). Similarly, in September 2020, parents from a small village rushed to protest at their local school after a child allegedly told their parents about the “forced vaccination” of pupils against COVID-19 (15). One video showing the parental concerns and the scolding of school staff quickly gained millions of views on social media and was used extensively to justify opposition to vaccines.

Social media need not be detrimental to the prevention and control of infectious diseases. Indeed, if its potential can be harnessed efficiently, public health interventions may gain significant advantages. Fortunately, such is the case with vaccination in Romania, where social media platforms have become an important vector for the delivery of accurate scientific information. For example, Romania has the largest Facebook group in the world that provides parents with vaccination advice directly from primary-care physicians (16, 17). With this in mind, the use of such social media platforms will prove beneficial in addressing vaccine hesitancy in Romania, thereby aiding the implementation of any potential anti-COVID-19 immunization strategy.

Lastly, the success of any immunization campaign depends on the sociocultural and historical factors that characterize the region(s) of the concerned populations. Important contributions to national public health campaigns can be offered by non-governmental entities, as was demonstrated in the course of the COVID-19 pandemic. One of the most illustrative examples in Romania concerns the Romanian Orthodox Church, which contributed both by providing significant human and material resources and by reinforcing the messages that were communicated by public health authorities (5). This was not always the case, as was observed during important religious holidays, when mass gatherings and protests against social distancing were recorded (18). However, in early January 2021, the Patriarch of the Romanian Orthodox Church issued a statement addressed to clerical staff in which he officially endorsed vaccination whilst stressing the importance of an accurate and transparent provision of information. Furthermore, this message included an official brochure which was elaborated by public health officials in order to provide accessible details concerning COVID-19 immunizations (19). This illustrates the importance of establishing clear channels of communication that go beyond classical methods and informing society from different angles to guide collective actions toward the betterment of public health.

## Future Directions and Proposed Recommendations

In Romania, by the end of December 2020, there have been more than half a million documented SARS-CoV-2 infections, with the real numbers likely far exceeding this figure due to insufficient testing (20). Despite the restrictions that were imposed by the authorities, hospitals are likely to become overwhelmed by COVID-19 patients and their capacity to provide intensive care will likely reach its limits. This situation is extremely alarming and other, perhaps more restrictive, interventions will be required to prevent a potential sanitary disaster. However, in the long term, equally worrying problems need to be addressed concerning the employment of a COVID-19 immunization campaign. Indeed, many of the previous Romanian *ad-hoc* vaccination campaigns which were not based on a solid framework did not achieve their desired goals. Such is the case with the 2008–2009 HPV vaccination campaign, which had a staggeringly low success rate (2.57%) without facing the many difficulties associated with the COVID-19 pandemic (21).

As the Romanian healthcare system is under immense pressure, an evidence-based strategy drawing on implementation science is the most effective means of delivering such a task (22). A successful campaign needs to be based on methods that were validated in other countries, and this is indeed what the EU general recommendations are relying on (1, 23). Subsequently, such methods need to be adapted to the unique profile of Romania, thereby directly addressing any country-specific challenges. With this in mind, we argue that the following criteria need to be met to guarantee the success of a national COVID-19 immunization strategy:

- Public health actions in Romania must be taken based on the lessons learned from successful vaccination campaigns in other countries of the EU and around the world. The available scientific knowledge concerning the implementation of good practices should serve as a basis for thorough planning and informed choices with regards to country-specific issues.
- COVID-19 testing must continue, and the national testing capacity must be increased for accurate data to inform the vaccination campaign. Currently, Romania's testing strategy offers a limited insight into the number of daily infections. A better understanding of the incidence rate coupled with cross-sectional studies of seroprevalence will inform strategies which could be tailored to specific regions of the country.
- Potential issues concerning the logistics of transport and storage need to be addressed in a centralized approach, and current impediments to the delivery infrastructure need to be identified and tackled. The involvement of military personnel in this process may overcome most of these challenges.
- The present infrastructure and information network concerning immunizations needs to be continuously developed to meet the demands of COVID-19 vaccination. Some of the resources available presently have the potential to be adapted for future requirements, and the feasibility of such actions will need to be carefully evaluated.
- The locations and stakeholders involved in immunization need to be clearly defined. Moreover, the Romanian public needs to be informed about the responsibilities of each concerned party throughout the vaccine implementation process.
- The identification and immunization of the priority groups should be explained and done appropriately. At the same time, equitable access to vaccination must be guaranteed, and constant communication concerning this aspect will be required during the COVID-19 immunization campaign in Romania.
- The transparency of both decision-making and implementation coupled with efficient communication will be crucial for a national vaccination strategy to succeed and for people to accept immunizations.
- Mechanisms to report potential side-effects or other issues concerning immunization need to be carefully defined and communicated in a clear and transparent manner.
- An elaborate and evidence-based vaccine awareness campaign must be carried out as soon as possible to tackle both general vaccine hesitancy and any potential mistrust concerning the provisioning of healthcare services. Moreover, classical awareness and educational campaigns will be insufficient if not brought together through an optimally devised delivery strategy that can be tailored to different at-risk categories of the population.
- Raising awareness and managing misinformation must be carried out on all channels of communication in Romania, with social media being of particular importance. With this in mind, the involvement of various influencers on these platforms will be key. At the same time, communication experts, psychologists, and sociologists should be approached to tailor the messaging strategy to various age and risk groups, according to national or local behavioral patterns.
- The support of influential figures and non-governmental institutions and organizations is required to facilitate an increase in the public understanding and acceptance of vaccines.
- A unifying legislative framework about vaccination is necessary to define the duties and responsibilities of all stakeholders involved in immunization. This will not only provide a basis for any national vaccination strategy, but will also ensure that all potential concerns are addressed in a clear and transparent manner.

Together, these recommendations will create an efficient and sustainable vaccine implementation framework which will serve as a basis for tackling the current challenges posed by the COVID-19 pandemic in Romania.

## Conclusion

In this article, we have described some of the main issues concerning the COVID-19 vaccination campaign in Romania. We also highlighted mechanisms through which many of these difficulties can be overcome, provided certain criteria are satisfied and actions are taken in a timely manner. Although most of the challenges that we presented in this article are country-specific, the proposed solutions could be adapted in order to inform similar public health policy development in other EU member states and beyond.

## Data Availability Statement

The original contributions presented in the study are included in the article and further inquiries can be directed to the corresponding author.

## Author Contributions

SD, OC, and OG wrote the main text of the manuscript. SD coordinated the academic initiative which resulted in the drafting of this article. OC and OG contributed equally to the work. RC, GDi, GDu, VG, and EP provided very insightful suggestions and assistance throughout the drafting of the manuscript. All authors contributed to the article and approved the submitted version.

## Conflict of Interest

The authors declare that the research was conducted in the absence of any commercial or financial relationships that could be construed as a potential conflict of interest.
